# AZOrange - High performance open source machine learning for QSAR modeling in a graphical programming environment

**DOI:** 10.1186/1758-2946-3-28

**Published:** 2011-07-28

**Authors:** Jonna C Stålring, Lars A Carlsson, Pedro Almeida, Scott Boyer

**Affiliations:** 1 Computational Toxicology, Global Safety Assessment, AstraZeneca R&D, Pepparedsleden 1, 431 53 Mölndal, Sweden; 2 EngInMotion Ltd, Avenida Infante D. Henrique, n. 145, 3510-070 Viseu, Portugal

## Abstract

**Background:**

Machine learning has a vast range of applications. In particular, advanced machine learning methods are routinely and increasingly used in quantitative structure activity relationship (QSAR) modeling. QSAR data sets often encompass tens of thousands of compounds and the size of proprietary, as well as public data sets, is rapidly growing. Hence, there is a demand for computationally efficient machine learning algorithms, easily available to researchers without extensive machine learning knowledge. In granting the scientific principles of transparency and reproducibility, Open Source solutions are increasingly acknowledged by regulatory authorities. Thus, an Open Source state-of-the-art high performance machine learning platform, interfacing multiple, customized machine learning algorithms for both graphical programming and scripting, to be used for large scale development of QSAR models of regulatory quality, is of great value to the QSAR community.

**Results:**

This paper describes the implementation of the Open Source machine learning package AZOrange. AZOrange is specially developed to support batch generation of QSAR models in providing the full work flow of QSAR modeling, from descriptor calculation to automated model building, validation and selection. The automated work flow relies upon the customization of the machine learning algorithms and a generalized, automated model hyper-parameter selection process. Several high performance machine learning algorithms are interfaced for efficient data set specific selection of the statistical method, promoting model accuracy. Using the high performance machine learning algorithms of AZOrange does not require programming knowledge as flexible applications can be created, not only at a scripting level, but also in a graphical programming environment.

**Conclusions:**

AZOrange is a step towards meeting the needs for an Open Source high performance machine learning platform, supporting the efficient development of highly accurate QSAR models fulfilling regulatory requirements.

## Background

Machine learning is applied within a vast range of disciplines such as economical forecasting, robotics, image analysis and risk assessment. Scientists using machine learning are not in general machine learning experts themselves and the algorithmic understanding for the various methods could be rather limited. Additionally, within many of these disciplines, low level programming knowledge is not abundant and scientist are often restricted to predefined machine learning protocols, wrapped in some graphical environment.

The new European chemical legislation, REACH, requires the chemical industry to provide information on, for example, ecotoxicity and human safety for chemicals used on the European market. However, the legislation does not support an increased usage of laboratory animals, but rather advocates the sharing of data and the development of alternative in vitro and in silico methods. Machine learning is routinely used in the prediction of chemical properties based on molecular structure information, so called quantitative structure activity relationships (QSARs). Hence, the ratification of the REACH legislation emphasizes the need to develop new methods for building and validation of QSAR models. The increased importance of QSAR modeling is manifested by the establishment of the OECD (Q)SAR project in 2004. The project aims to promote regulatory acceptance of QSAR approaches and it is in the process of establishing the "OECD Principals for the Validation for Regulatory Purpose of QSAR Models" [1].

Economical necessities and the concern for laboratory animals have driven the pharmaceutical industry in the same direction, replacing in vivo studies with in vitro experiments and in silico methods. Hence, QSAR modeling is becoming increasingly important also within drug discovery [2]. Information related to Absorption, Distribution, Metabolism, Excretion and Toxicity (ADMET) is relevant to all pharmaceutical projects and the aim is often to build models intended to be applicable within vast ranges of chemical space. In developing such global models, as much of chemical diversity as possible is included in the training sets, often encompassing tens of thousands of compounds obtained from proprietary internal databases [3].

Several studies have shown that it is not possible to identify a single machine learning algorithm which will be the most accurate for all data sets, even restricted to QSAR applications [4]. Hence, a data set specific choice of modeling algorithm and perhaps also the usage of combined model predictions, has the potential of increasing the model applicability beyond what is achievable with a single algorithm [3]. Multivariate linear modeling algorithms, such as Partial Least Squares (PLS), are well established within the QSAR community. However, the often non-linear relationship between descriptors and biological responses is recognized and the application of non-linear machine learning algorithms for QSAR modeling is increasing [5,6]. In general, non-linear machine learning algorithms represent a more complex optimization problem than linear methods and therefor require more training examples. Hence, the non-linear machine learning methods are of particular interest for global QSAR modeling, for which they need to be used in conjunction with thorough statistical validation and assessment of the applicability domain. In addition, non-linear methods are considered more difficult to use because of the tweaking of model hyper-parameters, such as the number of hidden neurons in an artificial neural network, often required to build accurate models.

Commercial mathematical packages, for example MATLAB, interfaces several machine learning algorithms, while Simca and TreeNet are commercial packages, well established within the QSAR community, thought developed around a single modeling algorithm. The Open Source statistical package R [7] has several third party modules with state-of-the-art machine learning methods. Orange [8] and Weka [9] are developed to be machine learning platforms providing multiple algorithms together with preprocessing and validation methods, while Knime [10] is a general pipe-lining system with several machine learning plug-ins.

Model hyper-parameter selection aims to find the parameters with the greatest generalization accuracy for a given data set by comparing the accuracy for different combinations of hyper-parameters. A few machine learning packages implement semi-automated model hyper-parameter selection. The Random Forest (RF) module of R monotonously increases the number of active variables until the out-of-bag (OOB) error no longer decreases. The libSVM [11] authors recommend a grid search to find the *C *and *γ *parameters with the best cross validation (CV) accuracy, while the grid search in the OpenCV [12] SVM implementation can optimize the *C*, *γ*, *p*, *nu*, *coef f *and *degree *parameters. Weka offers the possibility to calculate the CV accuracy varying a single parameter within a user defined interval. In addition, Weka implements a grid search restricted to two model parameters.

Despite the diversity of available machine learning packages, there is no package fulfilling all of the requirements on an Open Source state-of-the-art QSAR modeling platform. Such a system needs to include all tools necessary within a work flow encompassing database communication, data preprocessing, descriptor calculation and selection, model building and validation. It should be possible to build flexible machine learning applications in a graphical programming environment, as well as in a scripting mode. Because data sets often contain tens of thousands of compounds and the size of available data sets is expected to grow rapidly, the machine learning algorithms need to be highly numerically efficient. To exhaust the statistical aspects of model development, multiple and complementary machine learning algorithms should be made available. Complex modeling algorithms need to be customized for non-expert users and model hyper-parameters selected in an automated work flow to increase accuracy and efficiency in the model development process. Finally, the system should make it easy to develop models compliant with the OECD principals for validation of QSAR models.

AZOrange is a general Open Source platform for machine learning, however developed to meet the increasing demand for ADMET models in drug discovery in particular. AZOrange customizes several high performance state-of-the-art machine learning algorithms. The automated and generalized model hyper-parameter selection is a unique feature of AZOrange. The customization and the automated model hyper-parameter selection provide the tools necessary for automated model development, batch generation of models and the assessment of multiple model hypothesis. In addition, a graphical programming environment makes development of flexible high performance machine learning applications possible without scripting requirements.

## Implementation

The Open Source foundation of AZOrange gives complete algorithmic transparency, allows further development of the algorithms and reduces license costs. Furthermore, the Open Source solution grants the fundamental scientific principal of reproducibility, which is recognized in the OECD principals for QSAR modeling as an advantage over commercial packages. Making AZOrange itself an Open Source code reaches out to a larger group of users, thereby assuring a more extensive validation of the code.

The "Architecture" subsection describes the AZOrange architecture and the major Open Source dependencies, while the "Extension of Orange functionality" subsection gives a detailed overview of the functionality by which AZOrange complements the Orange package to facilitate ADMET modeling in particular.

### Architecture

**Figure 1:**
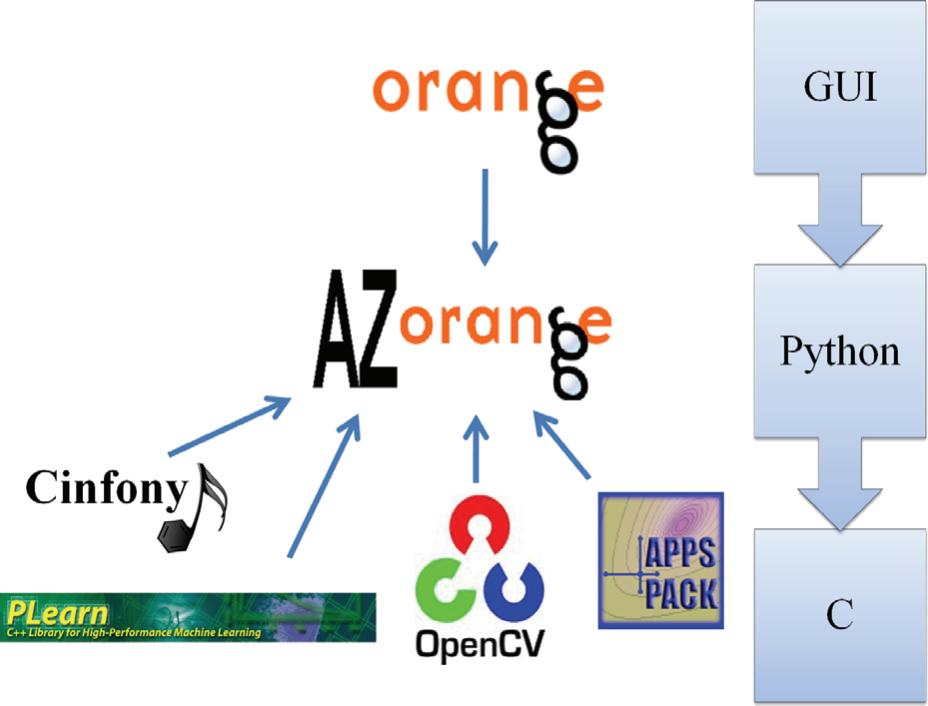
**The architecture of AZOrange**. The architecture and the major Open Source codes constituting AZOrange.

Because of its diversity, quality and architecture, AZOrange uses the Orange machine learning platform as a foundation. Orange implements the demanding numerical computations in C, while wrapping the top level objects in a Python scripting environment, as illustrated in Figure 1. The Python application programming interface (API) is used in a graphical user interface (GUI), providing a highly flexible framework for tailored machine learning application development. AZOrange interfaces Orange with a set of other Open Source codes to extend its functionality, in particular for QSAR modeling. The OpenCV package [12] adds a set of computationally efficient, non-linear machine learning algorithms. Although non-linear machine learning algorithms usually results in more accurate models for large descriptive QSAR data sets, a linear method constitutes a baseline. The PLearn [13] interface makes a partial least squares (PLS) algorithm executable from within the AZOrange framework. APPSPACK [14] was integrated for automated derivative free optimization of the model hyper-parameters, while Cinfony [15] provides AZOrange with a set of publically available molecular descriptors.

### Extension of Orange functionality

The major interfaces of AZOrange extend the functionality of Orange by incorporating descriptor calculation, additional persistent learners and generalized, automated model hyper-parameter selection. Further modifications are made to enhance feature ranking, prediction of external test sets and model persistency.

#### Molecular Descriptors

As AZOrange is intended to be a complete platform for QSAR modeling, a set of Open Source molecular descriptors is interfaced. Provided with SMILES, AZOrange calculates any descriptor within the Cinfony package and makes them available in Orange data objects. Cinfony is a mutual Python API for CDK [16], RDkit [17] and Open Babel [18], thereby efficiently interfacing the descriptors of these packages with AZOrange.

#### Feature ranking and selection

The Orange methods available for global ranking of features have been extended by the Random Trees (RT) variable importance assessment method [19] in OpenCV. The OpenCV implementation randomly permutes the values of one variable within the out-of-bag (OOB) set of examples of each tree. The OOB error of all trees, with and without permuted values, is used to quantify the importance of each variable. This RT variable importance assessment can be used to rank the importance of variables in a data set and consecutively in a wrapper variable selection algorithm.

#### Learners

The Orange learners are complemented by five new learners. These learners are implemented to comply with the Orange learner object standard and encompasses all functionality of these objects. The integrated learners are customized versions of the RT, Support Vector Machine (SVM), CvBoost and Artificial Neural Networks (ANN) implementations in OpenCV and the PLS algorithm in PLearn. The default model parameter values are those of OpenCV, but these values can be changed within AZOrange. All models except CvBoost, which is solely for binary classification, can be used with any dimensionality of the response variable. Furthermore, they are persistent, making AZOrange model predictions accessible from within other environments.

By default AZOrange imputes missing values with the average or the most frequent value of the training set, as implemented by the corresponding Orange method. Imputation is used on both the training set and on examples being predicted by AZOrange models. However, for Random Forest (RF) models, imputation can be replaced by defining surrogate nodes upon training, as originally proposed by Brieman [20]. The SVM and ANN algorithms require scaling of the variable values for the optimization algorithms to operate smoothly. Unless scaling is explicitly deselected, the ANN algorithm will use OpenCV functions to scale both the attribute values and the response variable. The OpenCV implementation of SVM does not have this inherent scaling. Hence, it is performed in AZOrange, transforming the variable values into the range between -1 and 1, using the same expression as in libSVM [11].

AZOrange implements a simple generalized consensus model, combining the predictions from AZOrange learners by averaging or by using the majority vote. A consensus prediction can be made even with an even number of classifiers if the individual classifiers calculate prediction probabilities. The class with the greatest sum of probabilities is predicted.

#### ANN customization

The OpenCV ANN algorithm is customized to reduce the risk for overfitting and to increase the chances of finding an optimal network. This is achieved by supporting early stopping based on the accuracy on a validation set [21] and by providing generalized methods for building multiple networks using different initial weights [22].

The ANN implementation in OpenCV supports two stopping criteria, reaching a predefined maximum number of epochs or a decrease in training set accuracy between two consecutive epochs (*ε*) below a user defined threshold. Using the *ε *criteria will stop the training when the first of these two criteria is met, while the maximum number of epochs disregards the change in training set accuracy.

The OpenCV stopping criteria have been complemented by an early stop criteria. When early stopping is used, 20% of the data will be selected by stratified random sampling to constitute a validation set, which is left outside of the updating of the weights. Lutz [21] examine three classes of early stopping criteria. For robustness with respect to noisiness on the accuracy surface, the third class of stopping criteria was selected. Hence, the accuracy is evaluated on the validation set every fifth epoch and the early stopping criteria is triggered when the performance does not improve over a user defined number of consecutive evaluations (defaulting to 5). The network with the best performance on the validation set is selected as the final model. When early stopping is enabled, the training of the network stops when the early stop criteria is triggered or when the maximum number of epochs is reached. The default maximum number of epochs has been increased to 3000.

The difficulty of finding the global minimum on any multi dimentional surface is well recognized, also in the context of optimization of the network weights of an ANN [22]. The chances of finding a more accurate network increases when training multiple networks while varying the initial weights, thus starting in different points on the surface. The initial weights in AZOrange are varied by controlling the seed of the pseudo random sampling in the Nguyen-Widrow initialization function used by OpenCV. The user can control the number of networks built and a final network is selected based on the accuracy on the validation set. The network resulting from the smallest number of iterations is selected when several networks have the same accuracy.

#### Model parameter selection

A general automated model parameter optimizer has been developed within AZOrange. Any number of parameters can be optimized simultaneously for the RF, SVM, ANN, CvBoost and PLS algorithms. For computational efficiency, the pattern search algorithm in APPSPACK is used to provide a derivative free search algorithm. Before starting the pattern search, the generalization accuracy is always assessed with the default model parameter configuration. Additionally, the mid point of each model parameter range is evaluated to provide an initial point for the pattern search. To reduce the risk of ending up in a local minimum, the pattern search can be complemented by an optional sparse grid search that could select an initial point other than the mid range point.

For model parameter selection purposes, the objective function needs to quantify the difference in generalization accuracy when varying the model parameter settings. Hence, an accurate generalization error is not critical, while correct relative generalization errors is paramount. The objective function used with the automated parameter optimizer is a double CV loop with any number of folds, however defaulting to a single 5-fold CV.

In an automated model parameter optimization scheme, special care should be taken to avoid overfitting as a result of the selection of too complex models. The generalization accuracy increases with increased model complexity up until a point where model flexibility can no longer be accounted for by the data set. Thus, this optimal model complexity is dependent on the size of the data set. Using a CV scheme to assess the generalization accuracy reduces the risk of overfitting, as compared to considering solely a training set accuracy. The tendency to select model parameters resulting in complex models could be moderated by introducing a regularization term, penalizing solutions with greater model complexity [23] or by considering the Akaike Information Criterion (AIC) [24]. The pragmatic approach controlling the parameter optimization in AZOrange thus far simply restricts the search intervals. Furthermore, the model parameter point with the greatest generalization accuracy could be disregarded if the improvement in accuracy is smaller than the variance originating from data sampling effects.

Multiple parameters control the architecture and complexity of machine learning algorithms. Even though the parameter optimizer handles any number of parameters simultaneously, a comprehensive optimization would in general be far too computationally expensive. Hence, for each machine learning algorithm, the parameters with the greatest impact on model accuracy need to be identified. Table 1 displays the parameters of the AZOrange machine learning algorithms selected for optimization by default. The ranges within which the parameters are optimized are also specified. The selection is supported by experience and results from literature. However, a more comprehensive study on the improvements in generalization accuracy upon optimizing various model parameters would be desirable.

**Table 1:** Optimized model hyper-parameters

Algorithm	Parameter	Range
RF	The number of active attributes	
SVM	C	2^-5 ^to 2^15^
	*γ*	2^-15 ^to 2^3^
ANN	The number of hidden neurons (one-layer)	
CvBoost	Maximum branching depth	1 to 20
	The number of trees	1 to 1000
PLS	The number of components	1 to *nAttr*-1

Model hyper-parameters being optimized by default and their corresponding ranges.

^1^*nAttr *is the number of attributes in the data set

^2^*nEx *is the number of examples in the data set

#### Miscellaneous

In addition to the major interfaces described above, AZOrange extends the functionality of Orange by various modifications to the Orange code.

AZOrange makes extensive use of automated model parameter selection to tune the machine learning algorithms for individual data sets. There is a clear risk of overestimating the generalization accuracy when the model hyper-parameters have been selected using the same data set. Hence, AZOrange has generalized methods to perform a double CV loop around the model parameter optimization. The generalization accuracy is assessed on the left out folds of the external loop, while model parameter optimization is performed on the corresponding training data, also using CV.

When a machine learning model is used for an extensive time period and new data is being made available during this time, it is important to be able to assess model performance on the new data. Alternatively, when there is a known time dependence in the data available at the time of developing the model, a temporal test set is a complement to other data sampling strategies used to assess the generalization accuracy of the model. Thus, AZOrange makes methods to quantify the performance on such separate test sets available in the GUI.

Methods for assessment of the applicability domain are crucial to a QSAR platform and an important area for further development. AZOrange includes a module for calculating the Mahalanobis distance in descriptor space between an example being predicted and the training set. The training set can either be represented by the nearest neighbors of the training set or the center of the set. An applicability domain can be estimated by considering the distribution of such Mahalanobis distances of compounds in an external test set. An example falling into the first quartile would be considered inside the applicability domain, while predictions of compounds in the last quartile would be considered unreliable. Using an external test set allows for assessment of the correlation between the Mahalanobis distance and the prediction error. The Mahalanobis distance based method already available within AZOrange is currently being complemented with multiple reliability methods in a collaborative effort with the Orange group. To enhance compatibility of Orange data objects while concatenating various data sets, the domain data objects are automatically harmonized. For example, type conversion is tried for variables with the same name but of different type and the order of enumeration variables is always forced to be aligned. The user is provided with information about the conversions required for compatibility. This domain compatibility enhancement is also applied to examples being predicted, for compliance with the model domain.

When developing a classification model it could be more important to have a high sensitivity for one class at the expense of a greater number of corresponding false predictions. Furthermore, classifiers could be biased and show a greater tendency to predict one of the classes, in particular for unbalanced data sets. In both these cases class weights can be used to shift the prediction distribution towards the desired class. The RF, SVM and ANN algorithms of AZorange implements support for weighting the importance of classes by setting the priors in the underlying OpenCV algorithm.

## Results and Discussion

The AZOrange package is intended to aid scientist with limited knowledge in machine learning to accurately use state-of-the-art machine learning algorithms. The customization of the interfaced learners includes, for example, descriptor scaling, optimization of model hyper-parameters and appropriate stopping criteria. Currently, AZOrange provides a framework by which fully automated QSAR pipelines can be constructed, while a generalized process is being developed. This process will accept a data set as the input and automatically return the most accurate model, selected amongst models based on all the statistical methods interfaced with Orange. To reduce the risk of overfitting and to avoid overestimating the generalization accuracy, the process under development uses multiple re-sampling of external test sets, for which no selection of methods or parameters has been done. In addition, the variation in accuracy between the folds in the external validation loop constitutes a stability criteria, also used in the model selection process. This process will further aid users with little experience in machine learning to build high quality QSAR models.

AZOrange is supported on the Ubuntu Lucid Lynx platform and freely accessible under the general public license (GPL) on a git hub. In addition, a version of the source code is provided together with this manuscript as "Additional file 1". The install script of AZOrange dynamically retrieves all dependencies from their repositories. The default installation process, documented in the README file of the git homepage, will automatically run the AZOrange test suite after installation.

This section gives a brief tutorial of the GUI, as well as references to example code for Python scripting with AZOrange. The examples are designed to cover functionality unique for AZOrange.

### Using the GUI

The GUI of Orange has been complemented by several widgets to enhance functionality important to make Orange a complete platform for predictive QSAR model development. The following Canvases will illustrate how to use some of the added methods.

#### Generalization accuracy with model parameter optimization

**Figure 2:**
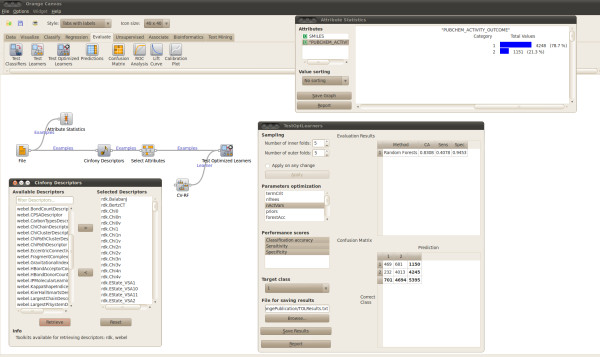
**Generalization accuracy**. Assessing the generalization accuracy of a learner with optimized model hyper-parameters with the double loop data sampling algorithm.

Figure 2 shows an AZOrange Canvas used to calculate RDK descriptors through the Cinfony widget for bioactivity data on the Estrogen receptor from PubChem (assay ID 639). The "Test Optimized Learners" widget is connected to the data set and to the AZOrange RF learner. Any model parameter can be selected for optimization and the number of folds in the inner and outer CV loops can be defined by the user. The presented accuracy metrics is the summation over the tests sets of the outer loop, while optimizing the selected model parameters using CV on the corresponding training sets.

#### Building and saving a model with optimized parameters

**Figure 3:**
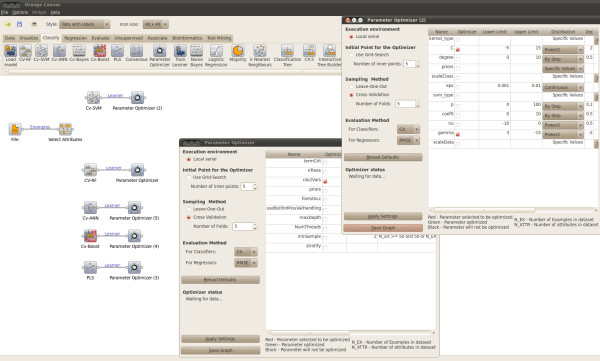
**Parameter optimization**. Using the "Parameter Optimizer" widget to optimize the parameters of any AZOrange learner.

**Figure 4:**
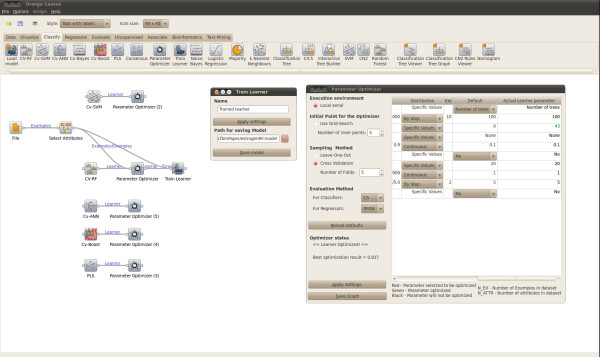
**Saving a model**. Saving a trained model with optimized model hyper-parameters.

Figure 3 and 4 show model parameter optimization and consecutive training of a model with the optimized parameters. In the "Parameter Optimizer" widget interface the sampling technique used to assess the generalization accuracy at each model parameter configuration can be selected. In addition, the mid range model parameter point used as the default initial point for the pattern search, can be replaced by the best point obtained with a grid search. The parameters displayed on the right hand side depend on the learner collected to the "Parameter Optimizer" widget. Figure 3 shows the optimizer interfaces while connected to the RF and SVM learners. The right hand side table defines the parameter default values, the allowed values and the ranges within which the parameters are optimized. The "Parameter Optimizer" widget returns an Orange learner object with optimized rather than default model parameters. The actual model is built by connecting the "Train Learner" widget with the "Parameter Optimizer" and the train data set. The "Train Learner" widget interface can be used to save the trained model to disk, as displayed in Figure 4.

#### Performance on external test sets

**Figure 5:**
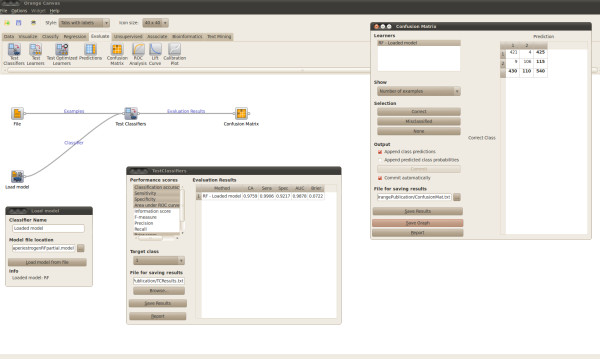
**External test set**. Test the performance of a saved model on an external test set.

Saved models can be loaded into AZOrange and used to predict temporal test sets as displayed in Figure 5. The temporal test set must contain all variables used while training the model. However, additional variables will be ignored upon predicting an example. Use the "Test Classifiers" widget to calculate accuracies of a trained model on the temporal data. "Test Classifiers" returns the Orange results object which can be used with for example the "Confusion Matrix" widget.

### Scripting with AZOrange

Even more flexible machine learning applications can be tailored using the Python scripting API of AZOrange. The basic principles of AZOrange scripting follow those of the Orange Python API. Three well commented scripts reproducing the work flows of the Canvases above, are available in the following sub directory of AZOrange: $AZORANGEHOME/doc/openExampleScripts

## Conclusions

AZOrange complements already available machine learning packages by interfacing and customizing several state-of-the-art machine learning algorithms. Multiple methods within the same package makes a data set specific selection of algorithm simple, potentially increasing accuracies beyond what is achievable in packages based on a single machine learning algorithm. The customization reduces the algorithmic knowledge requirements on users and allows users to concentrate on model development and data analysis, rather than programming and compatibility issues. For example, AZOrange transforms data formats, scales descriptor values where appropriate, accommodates missing values and selects stopping criteria. Model hyper-parameter selection is particularly important for non-linear machine learning algorithms and AZOrange optimizes any number of model hyper-parameters automatically and simultaneously for all its methods. This assures that a wider range of model parameters can be searched and makes the model development process more efficient as compared to manual tweaking of parameters. The AZOrange methods are accessible, not only at a scripting level, but also for development of flexible machine learning applications within a graphical user interface.

AZOrange is a complete platform for QSAR modeling, integrating data retrieval, descriptor calculation and selection, with training and validation. AZOrange is intended to aid in developing models compliant with the OECD principals for validation of QSAR models, by providing established methods for performance assessment and by granting the principal of transparency in being based solely upon Open Source codes. Furthermore, the tools for automated QSAR model development makes AZOrange suitable for large scale batch generation of QSAR models.

## Availability and Requirements

• Project name: AZOrange

• Project home page: http://github.com/AZcompTox/AZOrange

• Operating system: Ubuntu 10.04 LTS

• Programming language: C and Python

• Other requirements: All dependencies are automatically installed by the AZOrange installation procedure.

• License: GPL

## Competing interests

The authors declare that they have no competing interests.

## Authors' contributions

SB and LAC initially identified the need for the AZOrange platform and they explored the opportunities for using an Open Source solution. In addition, they identified the Orange package as a comprehensive machine learning tool already providing much of the desired functionality. JCS evaluated the computational efficiency of the OpenCV package and its potential to be used for QSAR applications. JCS, PA and LAC together explored the possibilities for using a pattern search algorithm for generalized and automated model hyper-parameter selection. JCS and PA have been responsible for the customization of the learners and the interface to the Cinfony package. JCS has been the main author of the manuscript, while all authors have contributed by revising and further developing the content.

## Supplementary Material

Additional file 1 **The AZOrange source code**. A zipped version of the AZOrange source code is provided. This version corresponds to the "chemistryCentral" tag in the git repository. Click here for file
