# Supplementary figures and images for: AZOrange - High performance open source machine learning for QSAR modeling in a graphical programming environment
Source: J Cheminform. 2011 Jul 28;3:28. doi: 10.1186/1758-2946-3-28 (PMC3158423; doi:10.1186/1758-2946-3-28)

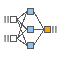

Supplement: Additional file 1 — The AZOrange source code. A zipped version of the AZOrange source code is provided. This version corresponds to the "chemistryCentral" tag in the git repository. [file 1758-2946-3-28-S1.ZIP › AZCompTox-AZOrange-528dda1/orange/OrangeWidgets/Classify/icons/ANN_60.png]

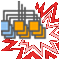

Supplement: Additional file 1 — The AZOrange source code. A zipped version of the AZOrange source code is provided. This version corresponds to the "chemistryCentral" tag in the git repository. [file 1758-2946-3-28-S1.ZIP › AZCompTox-AZOrange-528dda1/orange/OrangeWidgets/Classify/icons/Boost_60.png]

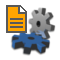

Supplement: Additional file 1 — The AZOrange source code. A zipped version of the AZOrange source code is provided. This version corresponds to the "chemistryCentral" tag in the git repository. [file 1758-2946-3-28-S1.ZIP › AZCompTox-AZOrange-528dda1/orange/OrangeWidgets/Classify/icons/LoadModel_60.png]

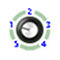

Supplement: Additional file 1 — The AZOrange source code. A zipped version of the AZOrange source code is provided. This version corresponds to the "chemistryCentral" tag in the git repository. [file 1758-2946-3-28-S1.ZIP › AZCompTox-AZOrange-528dda1/orange/OrangeWidgets/Classify/icons/Opt_60.png]

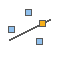

Supplement: Additional file 1 — The AZOrange source code. A zipped version of the AZOrange source code is provided. This version corresponds to the "chemistryCentral" tag in the git repository. [file 1758-2946-3-28-S1.ZIP › AZCompTox-AZOrange-528dda1/orange/OrangeWidgets/Classify/icons/PLS_60.png]

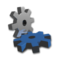

Supplement: Additional file 1 — The AZOrange source code. A zipped version of the AZOrange source code is provided. This version corresponds to the "chemistryCentral" tag in the git repository. [file 1758-2946-3-28-S1.ZIP › AZCompTox-AZOrange-528dda1/orange/OrangeWidgets/Classify/icons/Train_60.png]

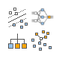

Supplement: Additional file 1 — The AZOrange source code. A zipped version of the AZOrange source code is provided. This version corresponds to the "chemistryCentral" tag in the git repository. [file 1758-2946-3-28-S1.ZIP › AZCompTox-AZOrange-528dda1/orange/OrangeWidgets/Classify/icons/consensus_60.png]

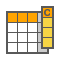

Supplement: Additional file 1 — The AZOrange source code. A zipped version of the AZOrange source code is provided. This version corresponds to the "chemistryCentral" tag in the git repository. [file 1758-2946-3-28-S1.ZIP › AZCompTox-AZOrange-528dda1/orange/OrangeWidgets/Data/icons/AddClass_60.png]

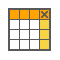

Supplement: Additional file 1 — The AZOrange source code. A zipped version of the AZOrange source code is provided. This version corresponds to the "chemistryCentral" tag in the git repository. [file 1758-2946-3-28-S1.ZIP › AZCompTox-AZOrange-528dda1/orange/OrangeWidgets/Data/icons/RenameClass_60.png]

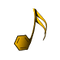

Supplement: Additional file 1 — The AZOrange source code. A zipped version of the AZOrange source code is provided. This version corresponds to the "chemistryCentral" tag in the git repository. [file 1758-2946-3-28-S1.ZIP › AZCompTox-AZOrange-528dda1/orange/OrangeWidgets/Data/icons/cinfony_60.png]

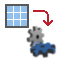

Supplement: Additional file 1 — The AZOrange source code. A zipped version of the AZOrange source code is provided. This version corresponds to the "chemistryCentral" tag in the git repository. [file 1758-2946-3-28-S1.ZIP › AZCompTox-AZOrange-528dda1/orange/OrangeWidgets/Evaluate/icons/TestClassifiers_60.png]

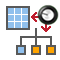

Supplement: Additional file 1 — The AZOrange source code. A zipped version of the AZOrange source code is provided. This version corresponds to the "chemistryCentral" tag in the git repository. [file 1758-2946-3-28-S1.ZIP › AZCompTox-AZOrange-528dda1/orange/OrangeWidgets/Evaluate/icons/TestOptLearners_60.png]
